# Life in the Living Room: An Expression of Personality in Late Life?

**DOI:** 10.1093/geroni/igaa057.2246

**Published:** 2020-12-16

**Authors:** Karen Fingerman, Yee To Ng, Shiyang Zhang

**Affiliations:** The University of Texas at Austin, Austin, Texas, United States

## Abstract

Recent research has found that micro environments (i.e., residential spaces) provide information about psychological processes in young adults. This study examined older adults’ living spaces. Participants (N = 171) from the Daily Experiences and Well-being Study completed measures of personality. Interviewers took 3 to 4 photographs of the room where participant spends the most time. Independent coders rated the rooms for 33 features (e.g., cleanliness, light, clutter) on a 5 point scale. Regression analyses revealed that personality was associated in anticipated ways. For example, conscientiousness was associated with rooms in better condition, neuroticism with less brightness, and by contrast, extraversion with greater brightness and more color. Discussion focuses on bidirectional emotional aspects of home environments that may contribute to well-being. Macro environments (e.g., neighborhoods) shape late life well-being, but understanding micro environments where older adults spend the majority of their time also may contribute to advances in the field.

